# Poly[[(μ_4_-5-amino­isophthalato)aqua­iron(II)] dihydrate]

**DOI:** 10.1107/S1600536808006326

**Published:** 2008-03-14

**Authors:** Wen-Dong Song, Jian-Bin Yan, Li-Li Ji, Hao Wang

**Affiliations:** a College of Science, Guang Dong Ocean University, Zhanjiang 524088, People’s Republic of China

## Abstract

In the title three-dimensional coordination polymer, {[Fe(C_8_H_5_NO_4_)(H_2_O)]·2H_2_O}_*n*_, the Fe^II^ atom exhibits a distorted octa­hedral geometry, being coordinated by one N and four O atoms from four 5-amino­isophthalate ligands and one water mol­ecule. In addition, the crystal structure is stabilized by numerous O—H⋯O and N—H⋯O hydrogen bonds.

## Related literature

For related literature, see: Wu *et al.* (2002 ▶); Zeng *et al.* (2007 ▶); Liao *et al.* (2006 ▶); Li *et al.* (2006 ▶).
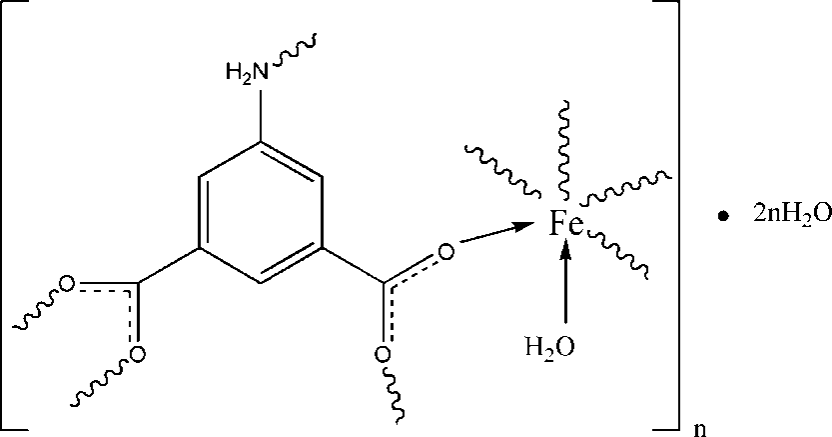


## Experimental

### 

#### Crystal data

[Fe(C_8_H_5_NO_4_)(H_2_O)]·2H_2_O
*M* *_r_* = 289.03
Triclinic, 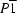

*a* = 7.7418 (2) Å
*b* = 8.5972 (2) Å
*c* = 8.6938 (2) Å
α = 85.560 (1)°
β = 76.058 (1)°
γ = 66.610 (1)°
*V* = 515.34 (2) Å^3^
*Z* = 2
Mo *K*α radiation
μ = 1.49 mm^−1^
*T* = 293 (2) K
0.20 × 0.18 × 0.17 mm

#### Data collection

Bruker APEXII area-detector diffractometer
Absorption correction: multi-scan (*SADABS*; Bruker, 2004 ▶) *T*_min_ = 0.755, *T*_max_ = 0.786
5025 measured reflections
2009 independent reflections
1895 reflections with *I* > 2σ(*I*)
*R*_int_ = 0.017

#### Refinement

*R*[*F* ^2^ > 2σ(*F* ^2^)] = 0.030
*wR*(*F* ^2^) = 0.090
*S* = 1.05
2009 reflections
172 parameters
11 restraints
H atoms treated by a mixture of independent and constrained refinement
Δρ_max_ = 0.37 e Å^−3^
Δρ_min_ = −0.57 e Å^−3^

### 

Data collection: *APEX2* (Bruker, 2004 ▶); cell refinement: *SAINT* (Bruker, 2004 ▶); data reduction: *SAINT*; program(s) used to solve structure: *SHELXS97* (Sheldrick, 2008 ▶); program(s) used to refine structure: *SHELXL97* (Sheldrick, 2008 ▶); molecular graphics: *SHELXTL* (Sheldrick, 2008 ▶); software used to prepare material for publication: *SHELXL97*.

## Supplementary Material

Crystal structure: contains datablocks I, global. DOI: 10.1107/S1600536808006326/gk2125sup1.cif

Structure factors: contains datablocks I. DOI: 10.1107/S1600536808006326/gk2125Isup2.hkl

Additional supplementary materials:  crystallographic information; 3D view; checkCIF report

## Figures and Tables

**Table 1:** Selected bond lengths (Å)

Fe1—O1	2.1040 (16)
Fe1—O2^i^	2.1364 (16)
Fe1—O1*W*	2.1458 (17)
Fe1—O4^ii^	2.2387 (17)
Fe1—O3^ii^	2.3416 (16)
Fe1—N1^iii^	2.376 (2)

Symmetry codes: (i) 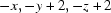; (ii) 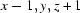; (iii) 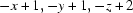.

**Table 2:** Hydrogen-bond geometry (Å, °)

*D*—H⋯*A*	*D*—H	H⋯*A*	*D*⋯*A*	*D*—H⋯*A*
O2*W*—H3*W*⋯O2*W*^iv^	0.844 (10)	2.377 (9)	2.892 (5)	119.9 (9)
O3*W*—H5*W*⋯O2^v^	0.843 (10)	2.09 (2)	2.852 (3)	151 (4)
O3*W*—H6*W*⋯O2*W*^vi^	0.840 (10)	2.071 (19)	2.865 (3)	157 (3)
O2*W*—H4*W*⋯O4^vii^	0.842 (10)	2.05 (2)	2.816 (3)	151 (3)
O1*W*—H2*W*⋯O2*W*^viii^	0.809 (9)	1.943 (12)	2.745 (3)	171 (3)
O1*W*—H1*W*⋯O3^ix^	0.815 (10)	1.914 (14)	2.705 (3)	163 (4)
N1—H1*B*⋯O3*W*	0.90	2.19	3.015 (3)	153

Symmetry codes: (iv) 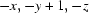; (v) 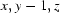; (vi) 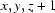; (vii) 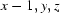; (viii) 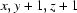; (ix) 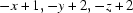.

**Fig. 1.**
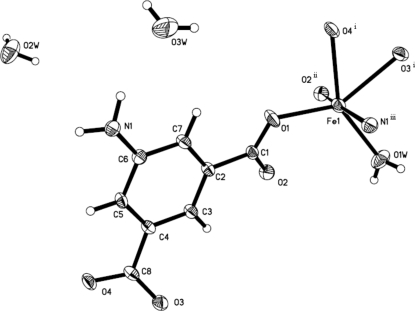
The structure of (I), showing the atom-numbering scheme and displacement ellipsoids drawn at the 50% probability level. H atoms have been omitted for clarity. [Symmetry codes: (i) -1 + x, y, z + 1; (ii) -x, 2 - y, 2 - z; (iii) 1 - x, 1 - y, 2 - z]]

**Fig. 2.**
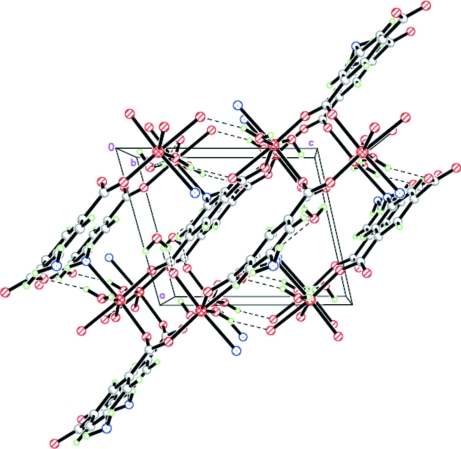
The three-dimensional network structure of the title compound, viewed along the b axis.

## supplementary crystallographic information

### Comment

5-Aminoisophthalatic acid is a good example of a bridging ligand that can link metal centres into extended networks, and a number of one-, two- and three- dimensional coordination frameworks have been generated (Zeng *et al.*, 2007; Wu *et al.*, 2002; Liao *et al.* 2006). Recently, we have obtained the title three-dimensional iron polymer, (I), and its crystal structure is reported here. This complex is isostructural with the Mn^II^ complex reported by Liao and Yao (2006) and by Li *et al.* (2006).

In the structure of (I) each Fe^II^ atom is coordinated by four O atoms from three 5-aminoisophthalate ligands, one N atom from another 5-aminoisophthalate ligand and one water molecule, and displays a distorted octahedral coordination geometry. The 5-aminoisophthalate ligands bridge iron ions to form a three-dimensional network (Fig. 2). Moreover, there are O—H···O and N—H···O hydrogen-bonding interactions within the three-dimensional structure connecting the carboxyl O atoms and amino N atoms of 5-aminoisophthalate ligands, the coordinating water molecules and water of crystallization (Table 2).

### Experimental

A mixture of FeCl_2_ (0.5 mmol), 5-aminoisophthalatic acid (0.5 mmol), NaOH (1 mmol) and H_2_O (12 ml) was placed in a 23 ml Teflon reactor, which was heated at 433 K for three days and then cooled to room temperature at a rate of 5 K h^-1^. Single crystals were obtained after washing with water and drying in air.

### Refinement

All H atoms attached to C and N atoms were fixed geometrically and treated as riding on their parent atoms with C—H = 0.93 Å (aromatic), N—H = 0.90 Å and *U*_iso_(H) = 1.2 *U*_eq_(C,*N*). H atoms from water molecules were located in difference Fourier maps and included in the subsequent refinement using restraints [O—H= 0.82 (1) Å and H···H= 1.34 (2) Å] with *U*_iso_(H) = 1.5*U*_eq_(O).

### Crystal data

**Table:**

[Fe(C_8_H_5_NO_4_)(H_2_O)]·2H_2_O	*Z* = 2
*M**_r_* = 289.03	*F*_000_ = 296
Triclinic, *P*1	*D*_x_ = 1.863 Mg m^−^^3^
Hall symbol: -P 1	Mo *K*α radiation λ = 0.71073 Å
*a* = 7.7418 (2) Å	Cell parameters from 1800 reflections
*b* = 8.5972 (2) Å	θ = 1.4–28.0º
*c* = 8.6938 (2) Å	µ = 1.49 mm^−^^1^
α = 85.560 (1)º	*T* = 293 (2) K
β = 76.058 (1)º	Block, red
γ = 66.610 (1)º	0.20 × 0.18 × 0.17 mm
*V* = 515.34 (2) Å^3^	

### Data collection

**Table:**

Bruker APEXII area-detector diffractometer	2009 independent reflections
Radiation source: fine-focus sealed tube	1895 reflections with *I* > 2σ(*I*)
Monochromator: graphite	*R*_int_ = 0.017
*T* = 293(2) K	θ_max_ = 26.0º
φ and ω scans	θ_min_ = 2.4º
Absorption correction: multi-scan(SADABS; Bruker, 2004)	*h* = −9→9
*T*_min_ = 0.755, *T*_max_ = 0.786	*k* = −10→10
5025 measured reflections	*l* = −10→10

### Refinement

**Table:**

Refinement on *F*^2^	Secondary atom site location: difference Fourier map
Least-squares matrix: full	Hydrogen site location: difference Fourier map
*R*[*F*^2^ > 2σ(*F*^2^)] = 0.030	H atoms treated by a mixture of independent and constrained refinement
*wR*(*F*^2^) = 0.090	*w* = 1/[σ^2^(*F*_o_^2^) + (0.0536*P*)^2^ + 0.5412*P*] where *P* = (*F*_o_^2^ + 2*F*_c_^2^)/3
*S* = 1.05	(Δ/σ)_max_ = 0.001
2009 reflections	Δρ_max_ = 0.37 e Å^−^^3^
172 parameters	Δρ_min_ = −0.57 e Å^−^^3^
11 restraints	Extinction correction: none
Primary atom site location: structure-invariant direct methods	

### Special details

**Table:**

Geometry. All e.s.d.'s (except the e.s.d. in the dihedral angle between two l.s. planes) are estimated using the full covariance matrix. The cell e.s.d.'s are taken into account individually in the estimation of e.s.d.'s in distances, angles and torsion angles; correlations between e.s.d.'s in cell parameters are only used when they are defined by crystal symmetry. An approximate (isotropic) treatment of cell e.s.d.'s is used for estimating e.s.d.'s involving l.s. planes.
Refinement. Refinement of *F*^2^ against ALL reflections. The weighted *R*-factor *wR* and goodness of fit *S* are based on *F*^2^, conventional *R*-factors *R* are based on *F*, with *F* set to zero for negative *F*^2^. The threshold expression of *F*^2^ > σ(*F*^2^) is used only for calculating *R*-factors(gt) *etc*. and is not relevant to the choice of reflections for refinement. *R*-factors based on *F*^2^ are statistically about twice as large as those based on *F*, and *R*- factors based on ALL data will be even larger.

### Fractional atomic coordinates and isotropic or equivalent isotropic displacement parameters (Å^2^)

**Table:**

	*x*	*y*	*z*	*U*_iso_*/*U*_eq_	
C1	0.2799 (3)	0.8073 (3)	0.9011 (2)	0.0165 (4)	
C2	0.4454 (3)	0.7019 (3)	0.7727 (2)	0.0161 (4)	
C3	0.5442 (3)	0.7791 (3)	0.6593 (2)	0.0172 (4)	
H3	0.5097	0.8957	0.6633	0.021*	
C4	0.6955 (3)	0.6798 (3)	0.5397 (2)	0.0168 (4)	
C5	0.7464 (3)	0.5058 (3)	0.5320 (2)	0.0178 (4)	
H5	0.8434	0.4414	0.4487	0.021*	
C6	0.6522 (3)	0.4277 (3)	0.6494 (2)	0.0167 (4)	
C7	0.5018 (3)	0.5267 (3)	0.7692 (2)	0.0178 (4)	
H7	0.4382	0.4755	0.8476	0.021*	
C8	0.8096 (3)	0.7607 (3)	0.4223 (2)	0.0178 (4)	
Fe1	0.02840 (4)	0.88499 (4)	1.21504 (3)	0.01967 (14)	
N1	0.7168 (3)	0.2480 (2)	0.6517 (2)	0.0204 (4)	
H1A	0.7573	0.2077	0.5513	0.025*	
H1B	0.6164	0.2203	0.6995	0.025*	
O1	0.2198 (2)	0.7325 (2)	1.01815 (18)	0.0251 (4)	
O2	0.2094 (2)	0.96615 (19)	0.88749 (19)	0.0208 (3)	
O3	0.8199 (3)	0.8942 (2)	0.46223 (19)	0.0243 (4)	
O4	0.8971 (2)	0.6932 (2)	0.28758 (18)	0.0252 (4)	
O1W	0.0523 (3)	1.1127 (2)	1.2728 (2)	0.0341 (4)	
H1W	0.112 (5)	1.105 (4)	1.340 (3)	0.051*	
H2W	0.065 (5)	1.189 (3)	1.217 (3)	0.051*	
O2W	0.0978 (4)	0.3865 (3)	0.1118 (3)	0.0480 (6)	
H4W	0.074 (5)	0.464 (4)	0.177 (4)	0.072*	
H3W	−0.001 (3)	0.401 (3)	0.078 (3)	0.072*	
O3W	0.4365 (3)	0.1532 (3)	0.9066 (3)	0.0482 (6)	
H5W	0.376 (5)	0.110 (5)	0.867 (5)	0.072*	
H6W	0.360 (4)	0.231 (4)	0.972 (4)	0.072*	

### Atomic displacement parameters (Å^2^)

**Table:**

	*U*^11^	*U*^22^	*U*^33^	*U*^12^	*U*^13^	*U*^23^
C1	0.0130 (10)	0.0204 (11)	0.0149 (10)	−0.0057 (9)	−0.0015 (8)	−0.0024 (8)
C2	0.0146 (10)	0.0162 (11)	0.0146 (9)	−0.0043 (9)	−0.0008 (8)	0.0006 (8)
C3	0.0171 (10)	0.0140 (10)	0.0172 (10)	−0.0045 (8)	−0.0007 (8)	0.0008 (8)
C4	0.0159 (10)	0.0189 (11)	0.0144 (9)	−0.0070 (9)	−0.0015 (8)	0.0023 (8)
C5	0.0155 (10)	0.0177 (11)	0.0152 (10)	−0.0034 (9)	0.0014 (8)	−0.0031 (8)
C6	0.0167 (10)	0.0141 (10)	0.0180 (10)	−0.0047 (9)	−0.0040 (8)	0.0008 (8)
C7	0.0171 (10)	0.0172 (11)	0.0162 (10)	−0.0065 (9)	0.0003 (8)	0.0020 (8)
C8	0.0143 (10)	0.0189 (11)	0.0164 (10)	−0.0045 (9)	−0.0015 (8)	0.0046 (8)
Fe1	0.0206 (2)	0.0162 (2)	0.0187 (2)	−0.00647 (15)	0.00079 (13)	−0.00049 (12)
N1	0.0215 (10)	0.0140 (9)	0.0234 (9)	−0.0064 (8)	−0.0008 (7)	−0.0024 (7)
O1	0.0263 (9)	0.0215 (8)	0.0176 (8)	−0.0064 (7)	0.0075 (6)	0.0003 (6)
O2	0.0173 (8)	0.0148 (8)	0.0255 (8)	−0.0027 (6)	−0.0020 (6)	−0.0015 (6)
O3	0.0296 (9)	0.0217 (8)	0.0216 (8)	−0.0140 (7)	0.0013 (7)	0.0010 (6)
O4	0.0289 (9)	0.0252 (9)	0.0169 (8)	−0.0124 (8)	0.0065 (6)	−0.0010 (6)
O1W	0.0553 (13)	0.0231 (9)	0.0353 (10)	−0.0223 (9)	−0.0204 (9)	0.0055 (7)
O2W	0.0719 (16)	0.0267 (10)	0.0453 (12)	−0.0136 (11)	−0.0229 (11)	−0.0003 (9)
O3W	0.0409 (12)	0.0553 (15)	0.0587 (14)	−0.0314 (11)	−0.0048 (10)	−0.0085 (11)

### Geometric parameters (Å, °)

**Table:**

C1—O1	1.254 (3)	C8—O3	1.262 (3)
C1—O2	1.262 (3)	Fe1—O1	2.1040 (16)
C1—C2	1.502 (3)	Fe1—O2^i^	2.1364 (16)
C2—C3	1.392 (3)	Fe1—O1W	2.1458 (17)
C2—C7	1.393 (3)	Fe1—O4^ii^	2.2387 (17)
C3—C4	1.392 (3)	Fe1—O3^ii^	2.3416 (16)
C3—H3	0.9300	Fe1—N1^iii^	2.376 (2)
C4—C5	1.390 (3)	N1—H1A	0.9000
C4—C8	1.499 (3)	N1—H1B	0.9000
C5—C6	1.396 (3)	O1W—H1W	0.815 (10)
C5—H5	0.9300	O1W—H2W	0.809 (9)
C6—C7	1.390 (3)	O2W—H4W	0.842 (10)
C6—N1	1.422 (3)	O2W—H3W	0.844 (10)
C7—H7	0.9300	O3W—H5W	0.843 (10)
C8—O4	1.256 (3)	O3W—H6W	0.840 (10)
			
O1—C1—O2	123.12 (19)	O1—Fe1—O4^ii^	90.79 (6)
O1—C1—C2	118.08 (19)	O2^i^—Fe1—O4^ii^	89.97 (6)
O2—C1—C2	118.80 (18)	O1W—Fe1—O4^ii^	148.03 (7)
C3—C2—C7	120.2 (2)	O1—Fe1—O3^ii^	145.97 (6)
C3—C2—C1	119.97 (19)	O2^i^—Fe1—O3^ii^	91.37 (6)
C7—C2—C1	119.81 (19)	O1W—Fe1—O3^ii^	90.96 (7)
C2—C3—C4	119.2 (2)	O4^ii^—Fe1—O3^ii^	57.10 (6)
C2—C3—H3	120.4	O1—Fe1—N1^iii^	85.72 (7)
C4—C3—H3	120.4	O2^i^—Fe1—N1^iii^	172.50 (6)
C5—C4—C3	120.64 (19)	O1W—Fe1—N1^iii^	83.48 (7)
C5—C4—C8	119.82 (19)	O4^ii^—Fe1—N1^iii^	94.39 (7)
C3—C4—C8	119.5 (2)	O3^ii^—Fe1—N1^iii^	85.91 (6)
C4—C5—C6	120.03 (19)	C6—N1—Fe1^iii^	113.51 (14)
C4—C5—H5	120.0	C6—N1—H1A	108.9
C6—C5—H5	120.0	Fe1^iii^—N1—H1A	108.9
C7—C6—C5	119.3 (2)	C6—N1—H1B	108.9
C7—C6—N1	120.25 (19)	Fe1^iii^—N1—H1B	108.9
C5—C6—N1	120.36 (19)	H1A—N1—H1B	107.7
C6—C7—C2	120.5 (2)	C1—O1—Fe1	116.73 (14)
C6—C7—H7	119.7	C1—O2—Fe1^i^	127.68 (14)
C2—C7—H7	119.7	C8—O3—Fe1^iv^	88.55 (12)
O4—C8—O3	120.90 (19)	C8—O4—Fe1^iv^	93.43 (13)
O4—C8—C4	119.9 (2)	Fe1—O1W—H1W	116 (2)
O3—C8—C4	119.15 (19)	Fe1—O1W—H2W	130 (2)
O1—Fe1—O2^i^	100.34 (7)	H1W—O1W—H2W	105.6 (16)
O1—Fe1—O1W	120.69 (8)	H4W—O2W—H3W	110.9 (17)
O2^i^—Fe1—O1W	89.59 (7)	H5W—O3W—H6W	111.2 (18)

Symmetry codes: (i) −*x*, −*y*+2, −*z*+2; (ii) *x*−1, *y*, *z*+1; (iii) −*x*+1, −*y*+1, −*z*+2; (iv) *x*+1, *y*, *z*−1.

### Hydrogen-bond geometry (Å, °)

**Table:**

*D*—H···*A*	*D*—H	H···*A*	*D*···*A*	*D*—H···*A*
O2W—H3W···O2W^v^	0.844 (10)	2.377 (9)	2.892 (5)	119.9 (9)
O3W—H5W···O2^vi^	0.843 (10)	2.09 (2)	2.852 (3)	151 (4)
O3W—H6W···O2W^vii^	0.840 (10)	2.071 (19)	2.865 (3)	157 (3)
O2W—H4W···O4^viii^	0.842 (10)	2.05 (2)	2.816 (3)	151 (3)
O1W—H2W···O2W^ix^	0.809 (9)	1.943 (12)	2.745 (3)	171 (3)
O1W—H1W···O3^x^	0.815 (10)	1.914 (14)	2.705 (3)	163 (4)
N1—H1B···O3W	0.90	2.19	3.015 (3)	153

Symmetry codes: (v) −*x*, −*y*+1, −*z*; (vi) *x*, *y*−1, *z*; (vii) *x*, *y*, *z*+1; (viii) *x*−1, *y*, *z*; (ix) *x*, *y*+1, *z*+1; (x) −*x*+1, −*y*+2, −*z*+2.
